# Glycyrrhinic acid and probiotics alleviate deoxynivalenol-induced cytotoxicity in intestinal epithelial cells

**DOI:** 10.1186/s13568-023-01564-5

**Published:** 2023-05-30

**Authors:** Xiaoxiang Xu, Juan Chang, Ping Wang, Chaoqi Liu, Ting Zhou, Qingqiang Yin, Guorong Yan

**Affiliations:** 1 Shanghai Skin Disease Hospital, School of Medicine, Tongji University, Shanghai, 200443 China; 2 College of Animal Science and Technology, Henan Agricultural University, Zhengzhou, 450046 China; 3 Guelph Research and Development Centre, Agriculture and Agri-Food Canada, Guelph, ON N1G 5C9 Canada

**Keywords:** Deoxynivalenol, Glycyrrhinic acid, Compound probiotics, Inflammation, IPEC-J2 cells

## Abstract

Deoxynivalenol (DON) is one of the most prevalent mycotoxin contaminants, which posing a serious health threat to animals and humans. Previous studies have found that individually supplemented probiotic or glycyrrhinic acid (GA) could degrade DON and alleviate DON-induced cytotoxicity. The present study investigated the effect of combining GA with *Saccharomyces cerevisiae* (*S. cerevisiae*) and *Enterococcus faecalis* (*E. faecalis*) using orthogonal design on alleviating IPEC-J2 cell damage induced by DON. The results showed that the optimal counts of *S. cerevisiae* and *E. faecalis* significantly promoted cell viability. The optimal combination for increasing cell viability was 400 µg/mL GA, 1 × 10^6^ CFU/mL *S. cerevisiae* and 1 × 10^6^ CFU/mL *E. faecalis* to make GAP, which not only significantly alleviated the DON toxicity but also achieved the highest degradation rate of DON (34.7%). Moreover, DON exposure significantly increased IL-8, Caspase3 and NF-κB contents, and upregulated the mRNA expressions of Bax, Caspase 3, NF-κB and the protein expressions of Bax, TNF-α and COX-2. However, GAP addition significantly reduced aforementioned genes and proteins. Furthermore, GAP addition significantly increased the mRNA expressions of Claudin-1, Occludin, GLUT2 and ASCT2, and the protein expressions of ZO-1, Claudin-1 and PePT1. It was inferred that the combination of GA, *S. cerevisiae*, and *E. faecalis* had the synergistic effect on enhancing cell viability and DON degradation, which could protect cells from DON-induced damage by reducing DON cytotoxicity, alleviating cell apoptosis and inflammation via inhibiting NF-κB signaling pathway, improving intestinal barrier function, and regulating nutrient absorption and transport. These findings suggest that GAP may have potential as a dietary supplement for livestock or humans exposed to DON-contaminated food or feed.

**Supplementary Information:**

The online version contains supplementary material available at 10.1186/s13568-023-01564-5.

## Introduction

Mycotoxins are a series of toxic secondary metabolites produced by fungi that frequently contaminate feed, cereal crops and foods worldwide, causing cell damage, sickness and even death for domestic animals, as well as cancer for human (Richard 2007). Insufficient understanding of mycotoxin contamination due to undeveloped mycotoxin detection technologies (Schelstraete et al. 2020), has led to a serious underestimation of harm to human health and animal production (Pitt and Miller 2017). According to the DSM World Mycotoxin Survey in 2021, deoxynivalenol (DON), fumitremorgin, and zearalenone are the most prevalent mycotoxin contaminants in raw cereal grains in China, with DON being the most common, accounting for 87% (https://www.biomin.net/solutions/mycotoxin-survey/). DON is a trichothecene B mycotoxin, also known as “vomitoxin” because of its emetic effect on organisms, especially swine. DON has acute and chronic toxicity, including cytotoxicity (Wang et al. 2016; He et al. 2021), immunotoxicity (Pestka and Smolinski 2005; Faeste et al. 2022), intestinal toxicity (Pinton and Oswald 2014; Huang et al. 2021). Animals exposed to DON usually suffer from nausea, vomiting, anorexia, abdominal pain, diarrhea and other symptoms (Mishra et al. 2020; Shen et al. 2021). Moreover, long-term exposure to DON can lead to immune suppression, malnutrition and slow growth. Therefore, it is crucial to develop effective measures to reduce DON residue in animals and mitigate the harm it causes. Developing an effective substance to prevent these damages is an urgent issue that needs to be addressed.

At present, there are a few methods available to achieve the safe and efficient detoxification of DON, nutritional regulation and probiotics being the most common ones. Glycyrrhinic acid (GA) is an extractive from glycyrrhiza that has proven to have anti-inflammatory, immunomodulatory, and anti-oxidative properties (Bentz et al. 2019; Afkhami-Poostchi et al. 2020; Akutagawa et al. 2019). Studies show that GA can improve the growth and meat quality of piglets (Alfajaro et al. 2012) and regulate autophagy to alleviate acute lung injury caused by lipopolysaccharides (Qu et al. 2019). In our previous study, we found that GA could alleviate DON-induced oxidative stress, inflammatory response and apoptosis through TNF and NF-κB signaling pathways in IPEC-J2 cells (Xu et al. 2020c). On the other hand, probiotics are considered as a substitute for antibiotics in farming, due to its benefits to gut barrier and immune system (Garcia et al. 2018). Our primary study has shown that the combination of compound probiotics with berberine could improve the health of piglets, enhance immunity, and reduce diarrhea rates (Xu et al. 2020d). Studies have reported that probiotics such as *Lactobacillus*, *Enterococcus faecalis* (*E. faecalis*), *Bifidobacteria* and *yeast*, as well as some compound probiotics, can effectively degrade mycotoxins (de Souza et al. 2020; Alassane-Kpembi et al. 2018). Our primary research also confirmed the alleviative effects of *Saccharomyces cerevisiae* (*S. cerevisiae*) in DON-induced inflammation (Chang et al. 2017). However, the combination effect of GA and compound probiotics in alleviating DON-induced cytotoxicity is still uninvestigated.

Overall, this study aimed to find the best combination and ratio of GA, *S. cerevisiae* and *E. faecalis* to effectively reduce the toxicity of DON in animal feed. By using an orthogonal design, researchers hope to optimize the compatibility of GA and probiotics to create a safer feed for animals. This study will provide useful information for the production of animal feed that is safe and healthy for consumption.

## Materials and methods

### Materials and reagents

DON (purity > 99%) was purchased from Sigma-Aldrich (St. Louis, MO, USA), and dissolved in dimethyl sulfoxide (DMSO) to obtain 1 mg/mL stock solution. GA was provided by Henan Delin Biological Products Co., Ltd., Xinxiang, China. DMSO, 0.25% pancreatin with ethylenediaminetetraacetic acid, phosphatebuffered saline (PBS), penicillin–streptomycin and thiazolyl blue tetrazolium bromide (MTT) were purchased from Solarbio (Beijing Solarbio Biotechnology Co., Ltd. Beijing, China). High-glucose Dulbecco’s Modified Eagle Medium (DMEM) and fetal bovine serum (FBS) were purchased from Biological Industries (Kibbutz Beit-Haemek, Israel). Yeast extract powder, tryptone, peptone, sodium chloride, glucose, methanol, anhydrous ethanol, potassium dihydrogen phosphate, anhydrous sodium acetate, manganese sulfate and magnesium sulfate were domestic analytically pure; DON quantitative detection kit was purchased from Suwei Biological Research Co., Ltd. Jiangsu, China. The IL-8, NF-κB, and Caspase 3 concentrations assay kits were purchased from Jiangsu Meimian Industrial Co., Ltd., Jiangsu, China. Rabbit polyclonal antibodies of Bax (abs119724), TNF-α (abs123966), COX-2 (abs120547), ZO-1 (abs131224), Claudin-1 (abs130064), PePT1 (abs134568), β-actin and goat anti-rabbit antibody of IgG were purchased from Absin Bioscience Inc. (Shanghai, China).

### Probiotics preparation

*Enterococcus faecalis (E. faecalis,* CGMCC1.2135*)* and Saccharomyces cerevisiae (*S. cerevisiae,* CGMCC 2.1542) used in the experiment were purchased from China General Microbiological Culture Collection Center (CGMCC), Beijing, China. *E. faecalis* and *S. cerevisiae* were incubated in MRS and YPD liquid media according to the previous report, respectively (Liu et al. 2019). The fermentation liquid of above probiotics were harvested after 36 h culture and determined by plating serial dilutions and measured as colony forming units (CFU), and then centrifuged at 8000 r/min for 5 min, the supernatant was absorbed, sterilized by 0.22 μm Minisart high-flow filter and stored at 4 °C for further use. The centrifuged cells were resuspended in equal volume using High-glucose DMEM medium without serum and antibiotics. The fermentation liquid, supernatant and cells were diluted to the different concentrations (viable counts of 1 × 10^2^, 1 × 10^3^, 1 × 10^4^, 1 × 10^5^ and 1 × 10^6^ CFU/mL) with High-glucose DMEM without serum and antibiotics.

### Cell culture

The cells were cultured in complete media, which comprised of High-glucose DMEM supplemented with 10% FBS and 1% penicillin–streptomycin in a humidified incubator at 37 °C with 5% CO_2_.

### Cell viability

IPEC-J2 cells were seeded into 96-well plate at a density of 1 × 10^4^ cells/ well (100 μL per well) and incubated for 24 h. Then the culture medium was removed, and the cells were washed twice with PBS. Next the cells were incubated with GA at concentrations of 50, 100, 200, 400 and 800 μg/mL, and the supernatant, cells and fermentation liquid of *E. faecalis* and *S. cerevisiae* were added at different concentrations of viable counts of 1 × 10^2^, 1 × 10^3^, 1 × 10^4^, 1 × 10^5^ and 1 × 10^6^ CFU/mL with or without 0.5 μg/mL DON for 6 h, respectively. GA and DON were diluted with High-glucose DMEM without serum and antibiotics. After all treatments, the cells were washed and incubated in serum-free media containing 0.5 mg/mL MTT at 37 °C with 5% CO_2_ for 4 h. Subsequently, the supernatant was removed, and each well was added with 150 μL DMSO and gently shaken for 15 min. The absorbance was measured at 490 nm with an ELx 800 microplate reader (BIO-TEK Instruments Inc., Winooski, VT, USA).

### Orthogonal experimental design and repeatability test validation

Based on the results of single-factor experiments, the viable count of *E. faecalis* and *S. cerevisiae,* and the concentration of GA were selected as experimental factors. L_9_ (3^4^) orthogonal design was selected to optimize the compound of the three substances. Here, L represented the orthogonal table; 9 was the total groups of experiment; 3 was the number of factors; 4 represented the maximum allowed number of factors. The design of factors and levels was shown in Table 1.

**Table 1:** Factors and levels of orthogonal experiment design

Levels	A GA (µg/mL)	B *S. cerevisiae* (lg, CFU/mL)	C *E. faecalis* (lg, CFU/mL)
1	200	4	4
2	400	5	5
3	600	6	6

### ELISA assay

IPEC-J2 cells were seeded at a density of 5 × 10^5^ cells/well in 6-well plate until the cell fusion rate reached 80%, and then incubated different treatments for 6 h. Thereafter, the cell supernatants of different treatments were collected and centrifuged at 12,000 rpm for 5 min. The concentration of IL-8, Caspase 3 and NF-κB were measured using enzyme-linked immunosorbent assays (ELISA) according to the manufacturer’s instructions. The absorbance was determined at 450 nm using an ELx 800 microplate reader (BIO-TEK Instruments Inc., Winooski, VT, USA).

### Quantitative real-time PCR and western blotting analysis

IPEC-J2 cells (5 × 10^5^ cells/well) were seeded in 6-well plate and allowed to culcure for 24 h, and then incubated four treatments for 6 h. Total RNA or protein were extracted with Trizol reagent (Takara) or RIPA buffer (EpiZyme Biotechnology, Shanghai, China) according to the manufacturer’s instructions, and then subjected to qRT-PCR or western blotting as previously described (Xu et al. 2020a). The detail primers were summarized in Additional file 1: Table S1.

### Statistical analysis

All data were expressed as mean ± standard deviation (SD). Differences between groups were determined by one-way ANOVA using SPSS 20.0 software, and Duncan’s multiple range test was used for multiple comparison. *P* < 0.05 indicates significant difference, while *P* > 0.05 indicates no significant difference.

## Results

### Effects of supernatant, cells and fermentation liquid of *S. cerevisiae* on cell viability in DON-induced IPEC-J2 cells

**Fig. 1:**
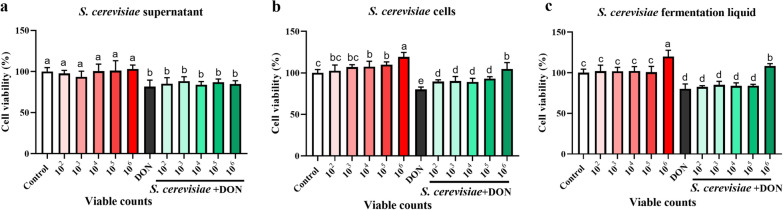
Effects of supernatant, cells and fermentation liquid of *S. cerevisiae* on cell viability. **a** Supernatant of *S. cerevisiae*; **b** Cells of *S. cerevisiae*; **c** Fermentation liquid of *S. cerevisiae*. All the values are expressed as the mean ± SD (n = 6). Different marked letters on each bar indicate significant difference from each other (*P* < 0.05), while the same marked letters on each bar indicate insignificant difference from each other (*P* > 0.05)

As shown in Fig. 1a–c, the supernatant, cells and fermentation liquid of *S. cerevisiae* had no toxicity to IPEC-J2 cells. Compared with the control group, the cell viability was significantly increased when the cells of *S. cerevisiae* were 1 × 10^4^, 1 × 10^5^ and 1 × 10^6^ CFU/mL (*P* < 0.05), and the supernatant of *S. cerevisiae* had no significant effect on cell viability (*P* > 0.05). In addition, compared with DON alone group, 1 × 10^6^ CFU/mL cells and fermentation liquid of *S. cerevisiae* addition could significantly increase cell viability (*P* < 0.05), while the supernatant had no significant effect (*P* > 0.05). Therefore, the cells of *S. cerevisiae* were selected as 1 × 10^4^, 1 × 10^5^ and 1 × 10^6^ CFU/mL in the subsequent experiments.

### Effects of supernatant, cells and fermentation liquid of *E. faecalis* on cell viability in DON-induced IPEC-J2 cells

**Fig. 2:**
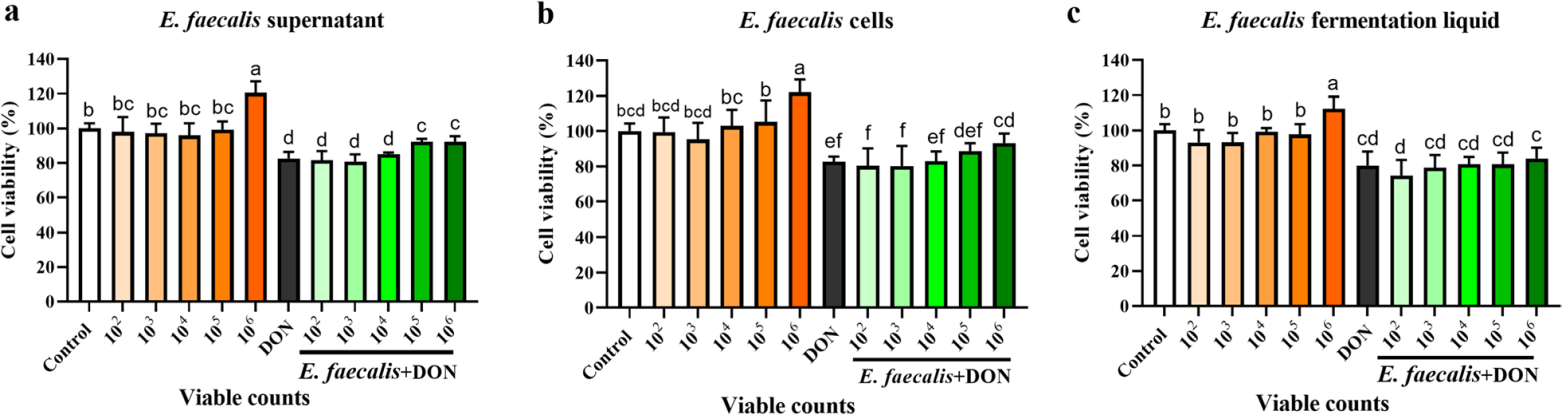
Effects of fermentation liquid, cells and supernatant of *E. faecalis* on cell viability. **a** Supernatant of *E. faecalis*; **b** Cells of *E. faecalis*; **c** Fermentation liquid of *E. faecalis*. All the values are expressed as the mean ± SD (n = 6). Different marked letters on each bar indicate significant difference from each other (*P* < 0.05), while the same marked letters on each bar indicate insignificant difference from each other (*P* > 0.05)

Figure 2a–c showed that the supernatant, cells and fermentation liquid of *E. faecalis* had no toxicity to IPEC-J2 cells, and the cell viabilities were significantly increased (*P* < 0.05) when the supernatant, cells and fermentation liquid of *E. faecalis* were 1 × 10^6^ CFU/mL, respectively, compared to the control group. Furthermore, compared with DON alone group, 1 × 10^6^ CFU/mL cells, 1 × 10^5^ and 1 × 10^6^ CFU/mL supernatant of *E. faecalis* additions could prominently enhance cell viability (*P* < 0.05). Hence, the cells of *E. faecalis* were selected as 1 × 10^4^, 1 × 10^5^ and 1 × 10^6^ CFU/mL for the subsequent orthogonal experiment.

### Effects of GA on cell viability

**Fig. 3:**
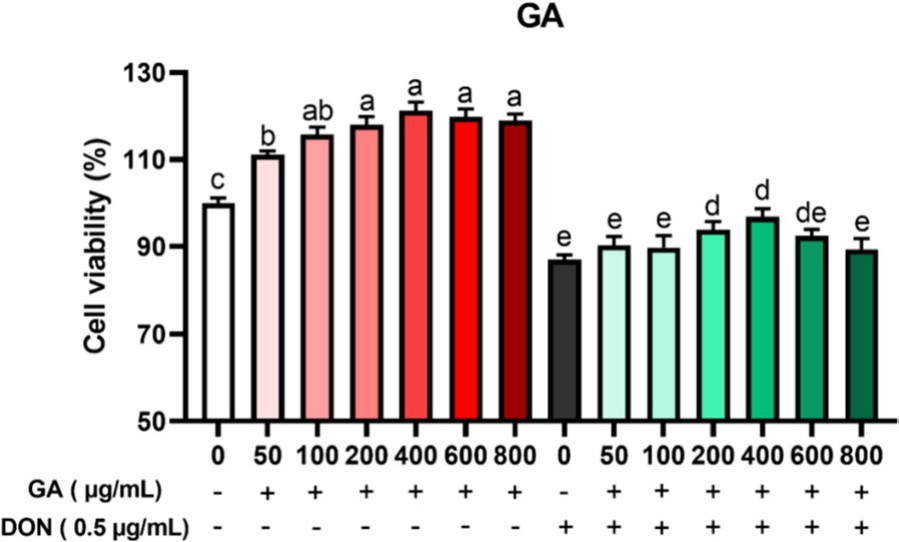
Effects of GA on cell viability. All the values are expressed as the mean ± SD (n = 6). Different marked letters on each bar indicate significant difference from each other (*P* < 0.05), while the same marked letters on each bar indicate insignificant difference from each other (*P* > 0.05)

Figure 3 showed that different concentrations of GA could significantly increase cell viability (*P* < 0.05), and the cell viability reached the maximum when GA concentration was 400 µg/mL, compared with the control group. Compared with DON alone group, 200 µg/mL and 400 µg/mL GA addition significantly increased cell viability. Therefore, 200, 400 and 600 µg/mL GA concentrations were selected for the subsequent orthogonal experiment.

### Optimization of *S. cerevisiae, E. faecalis* and GA on cell viability and DON degradation rate

According to the results of Additional file 1: Tables S2 and S3, the order of orthogonal factors in increasing cell viability was: B > A > C, and the order was changed to the following: B > C > A when the three factors were combined with DON; where A, B and C are the orthogonal factors representing GA*, S. cerevisiae* and *E. faecalis*. Three factors had no significant impact on cell viability (*P* > 0.05). The orthogonal experimental results in Tables 2 and 3 showed that the optimal level of combination was A2B3C1, indicating 400 µg/mL GA, 1 × 10^6^ CFU/mL *S. cerevisiae* and 1 × 10^4^ CFU/mL *E. faecalis*; while the best combination of range analysis was A2B3C3, indicating 400 µg/mL GA, 1 × 10^6^ CFU/mL *S. cerevisiae* and 1 × 10^6^ CFU/mL *E. faecalis*. Through verifying the above two results and their interactions (Table 4), it was found that the combination of 400 µg/mL GA, 1 × 10^6^ CFU/mL *S. cerevisiae* and 1 × 10^6^ CFU/mL *E. faecalis* could significantly increase the cell viability and alleviate the toxicity of DON (*P* < 0.05). In addition, the degradation rate of DON by this combination was 34.7%, which was significantly higher than that of other combinations (*P* < 0.05). Therefore, 400 µg/mL GA, 1 × 10^6^ CFU/mL *S. cerevisiae* and 1 × 10^6^ CFU/mL *E. faecalis* were selected as the optimal combination for the subsequent experiments.

**Table 2:** Effect of different combinations of GA*, S. cerevisiae* and* E. faecalis* on IPEC-J2 cell viability

Groups	A GA (µg/mL)	B *S. cerevisiae* (lg, CFU/mL)	C *E. faecalis* (lg, CFU/mL)	Cell viability (%)
Control	–	–	–	100.0 ± 2.2^de^
1	200	4	4	94.2 ± 1.7^f^
2	200	5	5	95.7 ± 2.3^ef^
3	200	6	6	110.0 ± 4.0^ab^
4	400	4	5	102.2 ± 2.5^cd^
5	400	5	6	106.5 ± 4.0^bc^
6	400	6	4	112.3 ± 4.1^a^
7	600	4	6	102.6 ± 6.5cd
8	600	5	4	106.7 ± 3.4^bc^
9	600	6	5	107.4 ± 4.5 ^b^
k1	99.9	99.1	104.4	
k2	107.0	103.0	101.2	
k3	105.6	109.9	106.4	

All the values are expressed as the mean ± SD (n = 6). Different marked letters in the column indicate significant difference from each other (*P* < 0.05), while the same marked letters in the column indicate insignificant difference from each other (*P* > 0.05)

**Table 3:** Effects of GA, *S. cerevisiae, E. faecalis* and DON synergies on IPEC-J2 cell viability

Groups	A GA (µg/mL)	B *S. cerevisiae* (lg, CFU/mL)	C *E. faecalis* (lg, CFU/mL)	Cell viability (%)
Control	–	–	–	100.0 ± 1.1^a^
DON	–	–	–	82.1 ± 2.4^de^
1	200	4	4	86.2 ± 4.1cd
2	200	5	5	83.8 ± 2.5^de^
3	200	6	6	90.6 ± 4.8^b^
4	400	4	5	86.6 ± 1.6^bc^
5	400	5	6	86.9 ± 1.7^bc^
6	400	6	4	91.3 ± 2.0^b^
7	600	4	6	90.3 ± 1.5^b^
8	600	5	4	87.0 ± 1.9^bc^
9	600	6	5	87.4 ± 2.7^bc^
k1	86.9	87.7	88.2	
k2	88.3	85.9	85.9	
k3	88.2	89.8	89.3	

All the values are expressed as the mean ± SD (n = 6). Different marked letters in the column indicate significant difference from each other (*P* < 0.05), while the same marked letters in the column indicate insignificant difference from each other (*P* > 0.05)

**Table 4:** Verification results of orthogonal and interactive effects of GA*, S. cerevisiae* and* E. faecalis* and their effects on DON degradation rate

Groups	Cell viability (%)	DON degradation rate (%)
Control	100.0 ± 1.5^d^	–
GA (400 µg/mL)	110.4 ± 3.4^cd^	–
*S. cerevisiae* (1 × 10^6^ CFU/mL)	115.3 ± 7.9^bc^	–
*E. faecalis* (1 × 10^6^ CFU/mL)	117.4 ± 5.2^bc^	–
*E. faecalis* (1 × 10^6^ CFU/mL)	100.6 ± 1.6^d^	–
GA + *S. cerevisiae* (1 × 10^6^ CFU/mL)	113.3 ± 6.6^bcd^	–
GA + *E. faecalis* (1 × 10^6^ CFU/mL)	116.8 ± 2.4^bc^	–
GA + *S. cerevisiae* (1 × 10^6^ CFU/mL) + *E. faecalis* (1 × 10^6^ CFU/mL)	125.9 ± 4.8^a^	–
GA + *S. Cerevisiae* (1 × 10^6^ CFU/mL) + *E. faecalis* (1 × 10^4^ CFU/mL)	121.3 ± 3.2^ab^	–
DON (0.5 μg/mL)	84.4 ± 2.1^de^	–
GA (400 µg/mL) + DON	90.4 ± 3.9^cde^	9.6 ± 2.1^f^
*S. Cerevisiae* (1 × 10^6^ CFU/mL) + DON	92.7 ± 3.2^bcd^	20.8 ± 0.9^d^
*E. faecalis* (1 × 10^6^ CFU/mL) + DON	93.8 ± 2.9^bc^	18.4 ± 1.1^de^
*E. faecalis* (1 × 10^4^ CFU/mL) + DON	87.2 ± 4.5^de^	16.5 ± 1.3^e^
GA + *S. Cerevisiae* (1 × 10^6^ CFU/mL) + DON	93.2 ± 1.9^bc^	28.3 ± 2.3^bc^
GA + *E. Faecalis* (1 × 10^6^ CFU/mL) + DON	96.1 ± 5.1^ab^	26.9 ± 1.7^c^
GA + *S. Cerevisiae* (1 × 10^6^ CFU/mL) + *E. faecalis* (1 × 10^6^ CFU/mL) + DON	100.1 ± 3.2^a^	34.7 ± 2.1^a^
GA + *S. Cerevisiae* (1 × 10^6^ CFU/mL) + *E. faecalis* (1 × 10^4^ CFU/mL) + DON	92.9 ± 3.9^bcd^	30.3 ± 1.7^ab^

“–” indicates undetermined. All values are expressed as the mean ± SD (n = 6). Different marked letters in the column indicate significant difference from each other (*P* < 0.05), while the same marked letters in the column indicate insignificant difference from each other (*P* > 0.05)

### Effects of GA and compound probiotics (GAP) on IL-8, Caspase 3 and NF-κB contents in DON-induced IPEC-J2 cells

**Fig. 4:**
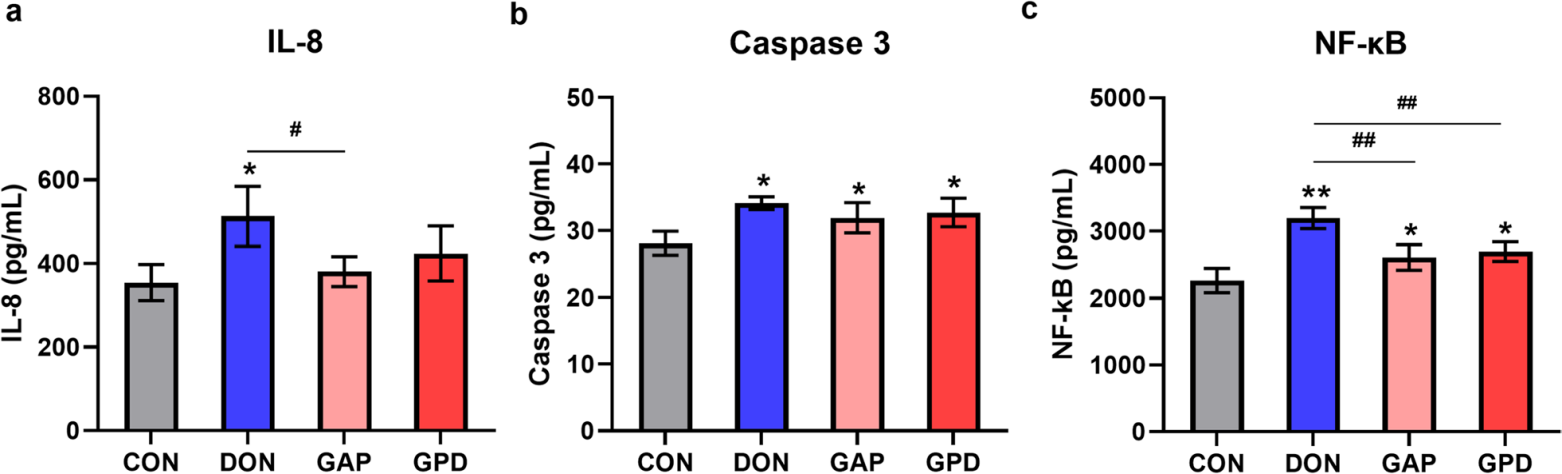
Effect of GAP on IL-8, Caspase 3 and NF-κB contents in IPEC-J2 cell supernatant induced by DON. **A**–**C**: IL-8, Caspase 3 and NF-κB contents in cell supernatant. CON: IPEC-J2 cells were treated with high glucose DMEM. DON: 0.5 μg/mL DON for 6 h. GAP: 400 µg/mL GA, 1 × 10^6^ CFU/mL *S. Cerevisiae* and 1 × 10^6^ CFU/mL *E. faecalis* for 6 h. GPD (GAP + DON): 400 µg/mL GA, 1 × 10^6^ CFU/mL *S. Cerevisiae*, 1 × 10^6^ CFU/mL *E. faecalis* and 0.5 μg/mL DON for 6 h. All the values are expressed as the mean ± SD (n = 3). Compared with the control group, *P < 0.05, **P < 0.01; compared with the DON group, ^#^*P* < 0.05, ^##^*P* < 0.01

As shown in Fig. 4a–c, compared with the control group, DON alone group significantly increased the contents of IL-8, Caspase 3 and NF-κB (*P* < 0.05). Compared to the DON alone group, GAP supplementation significantly decreased IL-8 and NF-κB contents (*P* < 0.05), GPD group significantly decreased the NF-κB content (*P* < 0.05), while there was no significant difference in the content of Caspase 3 (*P* > 0.05).

### Effects of GAP on apoptosis, tight junction protein and nutrient transport-related gene expressions in DON-induced IPEC-J2 cells

**Fig. 5:**
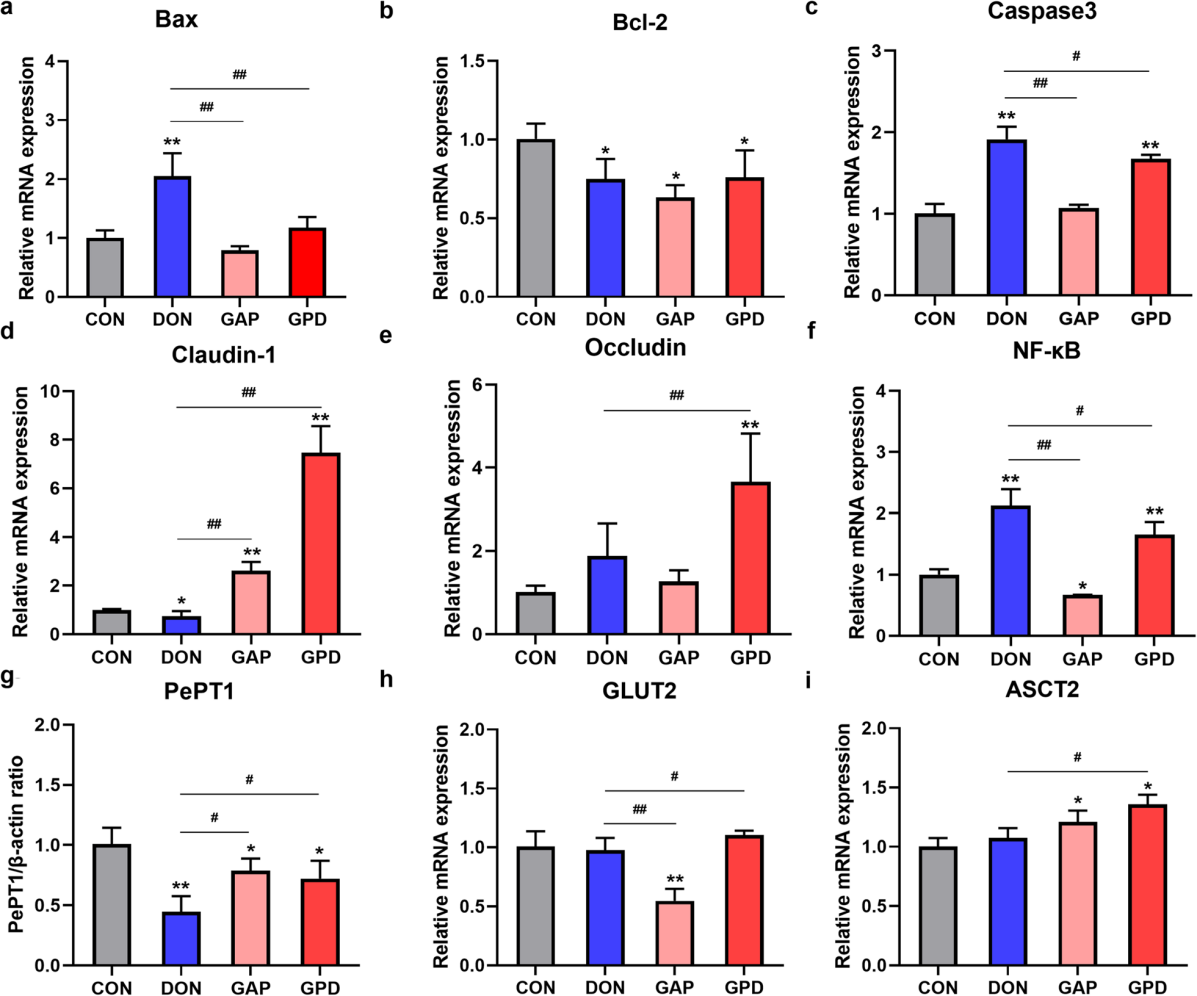
Effects of GAP on inflammation, apoptosis, tight junction protein and nutrient transport-related gene expressions in IPEC-J2 cells induced by DON. **a**–**i** Protein expressions of Bax, Bcl-2, Caspase 3, Claudin-1, Occludin, NF-κB, PePT1, ZO-1, GLUT2 and ASCT2. All the values are expressed as the mean ± SD (n = 3). Compared with the control group, *P < 0.05, **P < 0.01; compared with the DON group, ^#^*P* < 0.05, ^##^*P* < 0.01

It was shown that the relative mRNA abundances of Bax, Caspase 3 and NF-κB in the DON group were significantly upregulated, compared with the control group (*P* < 0.01); whereas they were significantly downregulated by GAP addition (*P* < 0.05). In addition, DON exposure remarkably downregulated the expressions of Bcl-2 and Claudin-1, compared with the control group (*P* < 0.01); while GPD group significantly upregulated the expressions of Claudin-1 and Occludin, compared with DON alone group (*P* < 0.05) (Fig. 5a–f). Figure 5g–i showed that DON alone group dramatically downregulated PepT1 expression compared with the control group (*P* < 0.05), while GAP addition significantly upregulated its expression (*P* < 0.05). Although there was no significant difference in the expressions of GLUT2 and ASCT2 between the DON alone group and control group, GPD group significantly increased their expressions (*P* < 0.05).

### Effects of GAP on inflammation, apoptosis, tight junction protein and nutrient transport-related protein expressions in DON-induced IPEC-J2 cells

**Fig. 6:**
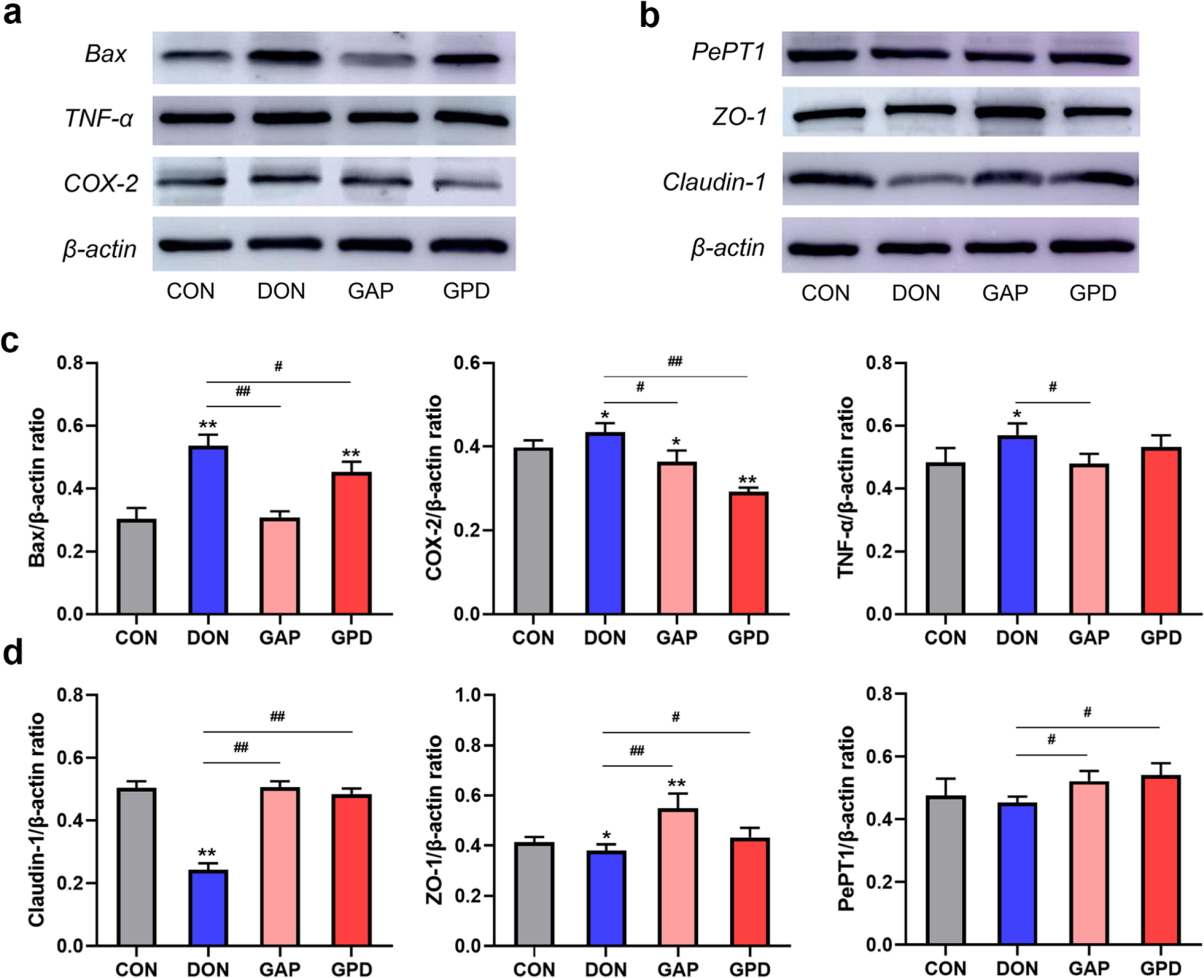
Effects of GAP on inflammation, apoptosis, tight junction protein and nutrient transport-related protein expressions in IPEC-J2 cells induced by DON. (**a** and **c**) Protein expressions of Bax, TNF-α and COX-2; (**b** and **d**) Protein expressions of ZO-1, Claudin-1 and PePT1. All the values are expressed as the mean ± SD (n = 3). Compared with the control group, *P < 0.05, **P < 0.01; compared with the DON group, ^#^*P* < 0.05, ^##^*P* < 0.01

The results in Fig. 6a–d indicated that compared with the control group, DON alone addition significantly increased the protein expressions of Bax, TNF-α and COX-2 (*P* < 0.05), and significantly decreased the protein expressions of ZO-1 and Claudin-1 (*P* < 0.05), but there was no significant difference in PePT1 protein expression (*P* > 0.05). Compared with DON alone group, GAP group significantly decreased the protein expressions of Bax, TNF-α and COX-2 (*P* < 0.05), and significantly increased the protein expressions of ZO-1, Claudin-1 (*P* < 0.01) and PePT1 (*P* < 0.05). The protein expressions of Bax and COX-2 were significantly decreased in GPD group (*P* < 0.05), while the protein expressions of ZO-1, Claudin-1 and PePT1 were significantly increased (*P* < 0.05).

## Discussion

The contamination of DON has caused extensive damage to animal healthy and production. In recent years, plant extracts and probiotics including yeast and lactic acid bacteria, exert an increasingly important role in the animal production. In the present study, the orthogonal design was adopted to optimize the ratio of GA, *S. cerevisiae*, and *E. faecalis* to obtain the best combination of these three substances to degrade DON and alleviate its cytotoxicity.

Probiotics have been widely used in livestock and poultry diets as good alternatives to antibiotics due to their prominent advantages of safety, non-pollution, and lack of residues (Pandey et al. 2015). Probiotics mainly have the characters of inhibiting the growth and reproduction of pathogenic bacteria in the gastrointestinal tract, strengthening the mucosal barrier, improving the function of the gastrointestinal tract, regulating the micro-ecological balance of the gastrointestinal tract, enhancing the immunity of the body, purifying the farming environment, degrading mycotoxins, and finally promoting animal production (Gaggia et al. 2010; Jha et al. 2020). Yeast and lactic acid bacteria are the most widely used probiotics as animal feed additives. Studies have shown that they can alleviate DON-induced porcine intestinal damage (Weaver et al. 2013; Ma et al. 2022; Maidana et al. 2021). At the same time, our previous research also found that *S. cerevisiae* has a certain repair effect on DON-induced IPEC-J2 cell damage, which can increase cell viability and protect cell integrity (Liu et al. 2019). Furthermore, *S. cerevisiae* was shown to protect against DON-induced inflammation by reducing the expression of downstream inflammatory cytokines and the activation of the p38 mitogen-activated protein kinase (p38 MAPK) pathway (Chang et al. 2017). *E. faecalis* is a facultative anaerobic gram-positive bacterium, which can improve animal growth performance, intestinal microflora, nutrient absorption and immunity (Thacker 2013; Maake et al. 2021; Zhang et al. 2019). In addition, *E. faecalis* exerts anti-inflammatory effects by modulating NF-κB, MAPK, and PPAR-γ1 pathways (Are et al. 2008; Oc et al. 2018). Previously, our team found that *E. faecalis* had a certain effect on degrading DON in vitro. In the present study, we investigated the effects of the supernatant, cells, and fermentation liquid of *S. cerevisiae* and *E. faecalis* on cell viability. The results showed that the microbes used in this study were non-toxic to cells, and a certain counts of viable bacteria (1 × 10^6^ CFU/mL) could significantly promote cell proliferation and reduce the toxic effects of DON. However, the effect of supernatant for cell viability was not significant. Research has shown that *Lc. paracasei LHZ-1* isolated from yogurt achieved a 40.7% reduction of DON by the cell wall. In contrast, only 10.5% and 8.9% were reduced by culture supernatant or cellular lysate, respectively (Zhai et al. 2019), which indicates that the supernatant of lactic acid bacteria for cell viability under DON treatment was limited. This study demonstrated that *S. cerevisiae* and *E. faecalis* have protective effects on cells.

As mentioned above, yeast and lactic acid bacteria play important roles in animal production, and their combination provides better benefits than individual addition. The interaction between mycotoxins and the functional groups of the cell surface results in mycotoxin adsorption on the cell wall structure. Yeast cell walls, which contain many different adsorption sites represented by polysaccharides, proteins, and lipids, play a crucial role in the detoxification process (Holanda et al. 2020; Faucet-Marquis et al. 2014). Since the mycotoxin adsorption is physical (based on ion exchange and complexation) (Huwig et al. 2001), mycotoxin contamination has been proven to bring little influence on yeast activity (Nathanail et al. 2016). Lactic acid bacteria, on the other hand, mainly rely on peptidoglycan and extracellular polysaccharide of the cell wall to adsorb toxins, thereby reducing the toxicity of mycotoxins. Our previous studies have indicated that GA can promote cell proliferation and reduce DON cytotoxicity (Xu et al. 2020c), and both *S. cerevisiae* and *E. faecalis* have certain effects on degrading DON. Therefore, the combination of compound probiotics and plant extracts could potentially have a higher efficacy in DON degradation and animal production. In this study, we optimized the combination of *S. cerevisiae*, *E. faecalis* and GA using an orthogonal experiment and explored the effects of this combination on the degradation of DON and alleviation of DON-induced cytotoxicity. The results showed that a certain amount of *S. cerevisiae* and *E. faecalis* could significantly promote IPEC-J2 cell proliferation, and there was a synergistic effect among different concentrations of *S. cerevisiae*, *E. faecalis* and GA. Specifically, the optimal efficiency was obtained under the combination of 400 µg/mL GA, 1 × 10^6^ CFU/mL *S. cerevisiae* and 1 × 10^6^ CFU/mL *E. faecalis*. This combination significantly improved cell viability, reduced the toxicity of DON, and maximized the degradation rate of DON. These findings are consistent with other studies that have demonstrated the significant increase in detoxification of mycotoxins with the combined use of compound probiotics compared to individual addition (Huang et al. 2018).

To further illuminate the alleviative mechanism of GAP on DON, we quantified changes in gene and protein expression related to inflammation, apoptosis, tight junction, and nutrient transport. Results revealed that DON significantly increased the contents of IL-8, Caspase3 and NF-κB, and upregulated the mRNA expressions of Bax, Caspase 3, NF-κB and the protein expressions of Bax, TNF-α and COX-2. However, GAP addition significantly reduced aforementioned genes and proteins, indicating that GAP might alleviate DON-induced inflammation and apoptosis by inhibiting of the NF-κB signaling pathway. In addition, DON exposure affected intestinal barrier function by downregulating ZO-1 and Claudin-1 proteins, whereas GAP significantly upregulated their expressions, which was in accordance with the previous report (Huang et al. 2019). Thus, we assume that the combination of GA and compound probiotics can alleviate the cytotoxicity induced by DON. Probiotics can reduce the damage caused by pathogens, drugs and other factors and increase intestinal tightness (Petrova et al. 2022). Compound probiotics can prevent cell inflammation and apoptosis by maintaining the stable expression of Claudin-1. In the present study, the combination of GA and compound probiotics increased the expression of Claudin-1, indicating that GAP could protect intestinal epithelial cells from DON damage. PepT1, GLUT2 and ASCT2 are the common and representative nutrient transporters. PePT1 is an oligopeptide transporter that mainly exists on the brush border membrane of small intestinal epithelial cells. It holds the function of transporting and absorbing dipeptide and tripeptide of protein degradation products, which plays an important role in maintaining the stability of the organism internal environment and the absorption of drugs in the gastrointestinal tract (Mertl et al. 2008). GLUT2 and ASCT2 were mainly responsible for glucose absorption and neutral amino acid transport of intestinal, respectively (Xu et al. 2020b). We found that GAP significantly increased the expression of PePT1, GLUT2 and ASCT2, which was beneficial to the transport and absorption of nutrients in the intestine, and alleviated the damage of DON to nutrient transport. The results revealed that GAP could enhance the intestinal barrier function and improve nutrient transport and absorption to mitigate the DON-induced cytotoxicity.

In conclusion, our study suggests that the combination of GA and compound probiotics can enhance the synergistic effect of cell viability and DON degradation, and protect IPEC-J2 cells from DON damage by reducing DON cytotoxicity and alleviating inflammation and apoptosis via inhibiting NF-κB signaling pathway, as well as improving intestinal barrier function and regulating nutrients transport and absorption. This study provides a theoretical basis for the acting mechanism of GA and compound probiotics as potential protective agents to reduce DON-induced cell damage, and also provides a reference for the use of GA and compound probiotics to prevent intestinal injury in humans and animals in the future.

## Supplementary Information

**Additional file 1: Table S1.** Primer sequences of genes for qRT-PCR. **Table S2.** Analysis of variance of GA, *S. cerevisiae* and *E. faecalis*. **Table S3.** Analysis of variance of orthogonal design for GA, *S. cerevisiae* and *E. faecalis* and DON synergies.

## Data Availability

All data generated or analyzed during this study are included in the present published article.

## Abbreviations

DON: Deoxynivalenol
GA: Glycyrrhinic acid
GAP: GA and compound probiotics
GPD: GAP and DON
DMSO: Dimethyl sulfoxide
PBS: Phosphate buffered saline
CFU: Colony forming units
DMEM: Dulbecco’s Modified Eagle Medium
FBS: Fetal bovine serum
SD: Standard deviation
