# Supplementary material for: Glycyrrhinic acid and probiotics alleviate deoxynivalenol-induced cytotoxicity in intestinal epithelial cells
Source: AMB Express. 2023 May 30;13:52. doi: 10.1186/s13568-023-01564-5 (PMC10229512; doi:10.1186/s13568-023-01564-5)
Supplement: Supplementary file 1 — Additional file 1: Table S1. Primer sequences of genes for qRT-PCR. Table S2. Analysis of variance of GA, S. cerevisiae and E. faecalis. Table S3. Analysis of variance of orthogonal design for GA, S. cerevisiae and E. faecalis and DON synergies. [file 13568_2023_1564_MOESM1_ESM.doc]

Additional file 1

Glycyrrhinic acid and probiotics alleviate deoxynivalenol induced cytotoxicity in intestinal epithelial cells

Xiaoxiang Xu^1^,^2^, Juan Chang^2^, Ping Wang^2^, Chaoqi Liu^2^, Ting Zhou^3^, Qingqiang Yin^2*^, Guorong Yan^1*^

^1^ Shanghai Skin Disease Hospital, School of Medicine, Tongji University, Shanghai 200443, China; xiaoxiangxu@stu.henau.edu.cn (X.X.), guorongyan@tongji.edu.cn (G.Y.).

^2^ College of Animal Science and Technology, Henan Agricultural University, Zhengzhou 450046, China; changjuan2000@henau.edu.cn (J.C.); wangping@henau.edu.cn (P.W.); liuchaoqi2018@henau.edu.cn (C.L.), qqy1964@henau.edu.cn (Q.Y.).

3 Guelph Research and Development Centre, Agriculture and Agri-Food Canada, Guelph, ON N1G 5C9, Canada; ting.zhou@agr.gc.ca (T.Z.)

^*^ Correspondence: qqy1964@henau.edu.cn (Q.Y.); guorongyan@tongji.edu.cn (G.Y.)

**Table S1 Primer sequences of genes for qRT-PCR**

| **Gene** | **Primer sequence (5’-3’)** |
| --- | --- |
| Bax | F: ATGATCGCAGCCGTGGACACG |
|  | R: AASTAGATGGTCACCGTCTGC |
| Bcl-2 | F: AGAGCCGTTTCGTCCCTTTC |
|  | R: GCACGTTTCCTAGASTGCAT |
| Caspase 3 | F: TTGGACTGTGGGATTGAGACG |
|  | R: CGCTGCACAAAGTGACTGGA |
| Occludin | F: ACCCAGCAAASTCATA |
|  | R: TCAASTTAAASTGCATA |
| Claudin-1 | F: ATTTCAGGTCTGGCTATCTTAGTTGC |
|  | R: AGGGCCTTGGTGTTGGGTAA |
| NF-κB | F: CTCGCACAAGGAGACATGAA |
|  | R: ACTCAGCCGGAAGGCATTAT |
| PePT1 | F: CAGACTTASTCCACAACGGA |
|  | R: TTATCCCGCCAGTACCCAGA |
| GLUT2 | F: ATTGTCACAGGCATTCTTGTTAGTCA |
|  | R: TTCACTTGATGCTTCTTCCCTTTC |
| ASCT2 | F: CTGGTCTCCTGGATCATGTGG |
|  | R: CAGGAAGCGGTAGGGGTTTT |
| GAPDH | F: ATGACCACAGTCCATGCCATC |
|  | R: CCTGCTTCACCACCTTCTTG |

**Table S2 Analysis of variance of GA*, S. cerevisiae* and *E. faecalis***

| **Source of variation** | **SS** | **df** | **MS** | **F value** | ***P* value** |
| --- | --- | --- | --- | --- | --- |
| A | 83.78 | 2 | 41.89 | 3.92 | 0.20 |
| B | 163.60 | 2 | 81.80 | 7.66 | 0.12 |
| C | 32.34 | 2 | 16.17 | 1.51 | 0.39 |
| Error | 21.37 | 8 | 10.68 |  |  |
| Total variation | 301.09 |  |  |  |  |
| R^2^ | 0.93 |  |  |  |  |

Note: MS is the mean square, SS is the sum of squared deviations, F is the F-statistic, and df is the degrees of freedom. A: GA, B: *S. cerevisiae*, C: *E. faecalis*.

**Table S3 Analysis of variance of orthogonal design for GA*, S. cerevisiae* and *E. faecalis*** and DON synergies

| Source of variation | SS | df | MS | F value | *P* value |
| --- | --- | --- | --- | --- | --- |
| A | 3.82 | 2 | 1.91 | 0.93 | 0.52 |
| B | 22.38 | 2 | 11.19 | 5.43 | 0.16 |
| C | 17.41 | 2 | 8.70 | 4.23 | 0.19 |
| Error | 4.12 | 8 | 2.06 |  |  |
| Total variation | 47.74 |  |  |  |  |
| R^2^ | 0.91 |  |  |  |  |

Note: MS is the mean square, SS is the sum of squared deviations, F is the F-statistic, and df is the degrees of freedom. A: GA, B: *S. cerevisiae*, C: *E. faecalis*.
